# A Short Breast Imaging Reporting and Data System-Based Description for Classification of Breast Mass Grade

**DOI:** 10.3390/life14121634

**Published:** 2024-12-09

**Authors:** Jonas Grande-Barreto, Gabriela C. Lopez-Armas, Jose Antonio Sanchez-Tiro, Hayde Peregrina-Barreto

**Affiliations:** 1 Tecnologías de la Información, Universidad Politécnica de Puebla, Cuanalá, Puebla 72640, Mexico; jonas.barreto385@uppuebla.edu.mx; 2 Centro de Enseñanza Técnica Industrial, C. Nueva Escocia 1885, Guadalajara 44638, Mexico; glopez@ceti.mx; 3 Instituto Nacional de Astrofísica, Óptica y Electrónica, Luis Enrique Erro 1, San Andres Cholula 72840, Mexico; jsantonio@inaoep.mx

**Keywords:** breast masses, BI-RADS grade, automatic classification, mass characterization

## Abstract

Identifying breast masses is relevant in early cancer detection. Automatic identification using computational methods helps assist medical experts with this task. Although high values have been reported in breast mass classification from digital mammograms, most results have focused on a general benign/malignant classification. According to the BI-RADS standard, masses are associated with cancer risk by grade depending on their specific shape, margin, and density characteristics. This work presents a methodology of testing several descriptors on the INbreast dataset, finding those better related to clinical assessment. The analysis provides a description based on BI-RADS for mass classification by combining neural networks and image processing. The results show that masses associated with grades BI-RADS-2 to BI-RADS-5 can be identified, reaching a general accuracy and sensitivity of 0.88±0.07. While this initial study is limited to a single dataset, it demonstrates the possibility of generating a description for automatic classification that is directly linked to the information analyzed by medical experts in clinical practice.

## 1. Introduction

Mammography is a comprehensive imaging tool for the early detection of breast cancer (BC), and several features like the density of the tissue [1], the presence of microcalcifications, and visualization of breast masses are essential to evaluate the risk of BC. According to GLOBOCAN data, by 2040, there will be about 3.16 million incidences of breast cancer, and mortality is estimated at 1 million women, making it the most common disease among women [2]. Health campaigns have focused on obtaining a mammogram after the age of 40, and the efforts in health education have not been sufficient in preventing and reducing deaths associated with BC. According to the Breast Imaging Reporting and Data System (BI-RADS), the diagnosis of BC with mammography is ranked into seven categories from 0 up to 6, with each number having a significance of being benign or malignant; four categories are also described as related to breast density (ACR-BI-RADS) [3] arranged from mostly fatty (ACR-A) tissue to mostly dense tissue (ACR-D). Breast density is one of the most critical features to verify on a mammogram, which does not allow the radiologist to visualize guide elements for categorizing the mammogram according to the BI-RADS [4]. Suppose the patient has a breast lesion belonging to categories BR-3 to BR-5, related to calcifications and mostly breast masses. In that case, the next step is to conduct a complementary study, such as an ultrasound with elastography or a biopsy, to determine the nature of the lesion.

The assessment and assignment of the BI-RADS and breast density by radiologists may constitute the worst situation that can occur in medical practice: a mass hidden in dense breasts if they do not have enough experience in categorizing mammograms or are experiencing work overburden. These are everyday situations in health centers; therefore, the development of emerging technologies that help radiologists quickly identify the characteristics associated with breast cancer could have a profound impact in the near future on this disease [5,6,7]. A study performed by Lameijer et al. [8] in 2021 found that a delay in diagnosis between 4 and 24 months tends to be more likely related to invasive cancer and that the main reason for the delay was an incorrect classification by the radiologist. Therefore, developing automatic systems for detecting breast lesions, such as masses in mammograms, provides a practical alternative to help support medical diagnoses [9].

In recent years, relevant contributions related to mass characterization using machine learning methods have been reported. Texture descriptors have often been used to analyze breast mass regions, as reported in [10], where a gray-level co-occurrence matrix (GLCM) was used to differentiate benign from malignant masses, reaching an accuracy of 94%. Khan et al. [11] used directional texture descriptors based on Gabor filters to identify masses (benign/malignant) and normal tissue. The best results were obtained using linear discriminant analysis (LDA), with an accuracy of 97%. In [12], the local photometric attributes (LPAs), local texture descriptors, were used to identify malignant masses, reaching a competitive accuracy (87%) compared to methods that use global texture filters. Breast mass form is also relevant since it can be analyzed with statistical and geometric measures, capturing invariant and noise-resistant characteristics [13,14]. In [15], several texture descriptors were used to identify if a region was suspicious as a mass or a nonmass with an accuracy of 92% and 95% on the MIAS and DDSM databases, respectively. Also, it specifically addressed the characterization of the shape of the mass, one of the features stated by the BI-RADS standard, to determine benignity/malignancy. A CAD system to identify abnormalities that could indicate benignity/malignity in breast masses with texture and shape features was developed in [16], reaching an accuracy of 94% for MIAS and 90% for DDSM. Recently, Singh et al. [17] explored feeding several classifiers with a mixture of previously selected textural and geometric characteristics. The results showed that an accuracy of 90.4% was reached by classifying benign and malignant masses by k-NN (k-nearest neighbors) and an exhaustive feature selection process. Sparse representations were also explored to perform mass characterization of benign/malignant masses [18]. The mammograms were divided into patches to build a dictionary with spatially localized ensemble sparse analysis (SLESA), achieving an accuracy of 90%.

Although these methods have achieved relevant improvements in benign/malignancy identification in breast masses, most analyses are neither based nor related to the BI-RADS characteristics, making it difficult for medical experts to use or interpret them. In general, the relevance of detecting breast masses is related to monitoring their evolution and taking preventive actions [5]. Thus, describing and quantifying standardized characteristics of breast masses is essential. Since the characteristics established by the -RADS are visually assessed, it is possible to relate them to descriptors of the digital image. In this work, it was hypothesized that quantifying breast mass features in mammograms could provide a suitable description that agrees with the BI-RADS standard and would be helpful for the radiologist in assessing benign/malignancy. Moreover, the utility of the proposed description was explored for specific identification of the BI-RADS classes, aiming for a graduated classification. The results were evaluated by an automatic classifier, reaching an accuracy of 0.90% in benign/malignancy classification and 87% in the specific identification of BI-RADS classes.

## 2. Materials and Methods

### 2.1. Masses Features in Terms of BI-RADS

**Figure 1:**
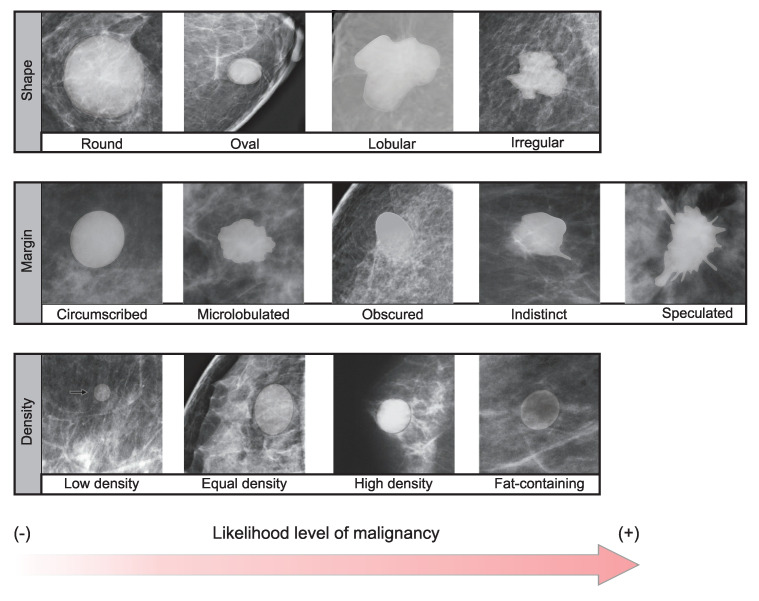
Characteristics associated with the benignity/malignancy of breast masses.

The BI-RADS standardizes the terminology and systematic of the mammography report, categorizing lesions according to the degree of suspicion of the findings [3]. Among the analyzed findings are the breast masses: the tridimensional lesions circumscribed in the tissue. If the radiologist detects a mass in the mammography, the next step is to determine its characteristics to establish a risk level or the need for more specific studies [19,20,21]. Masses are described by their morphology, margin, and density, as shown in Figure 1. A mass is more likely benign if it has a well-defined shape, circular or oval. Also, a contour (margin) observed to continue without abrupt changes (spicules) is associated with a benign mass. Finally, the density descriptor relates the inner density of the mass to the density of the tissue around it [22]; dense tissue appears shiny on a mammogram. Thus, these three characteristics are used to classify masses in the BI-RADS standard. For example, a mass with a round shape, circumscribed margin, and low density could be cataloged as a benign lesion (BR-2 or BR-3 in clinical terms). In contrast, a lobular mass with a speculated margin and high density could be diagnosed as a tumor (BR-4 or BR-5) [5].

### 2.2. Data Augmentation

Variations in scanners’ technical parameters generate gray-level variations in capturing tissues with similar characteristics. Aiming to consider this aspect of real mammograms, changes in mammogram contrast were induced, generating data augmentation. A gamma correction was used according to the intensity level of the image. To assess the intensity, the proportion of gray levels was computed with Equation (1), where $\mu_{I}$ y $\sigma_{I}$ corresponds to the mean and standard deviation of the mammogram and *L* to the highest value in the gray level. This ratio determines if the image has high or low intensity, allowing the contrast to change proportionally. After experimentation, ρ>70 was associated with brighter images. Then, gamma corrections of γ=0.5 and γ=0.3 were set for low and high-brightness images, respectively.

(1) $$\rho =100\times \frac{\left(\mu_{I}+\sigma_{I}\right)}{L}$$

**Figure 2:**
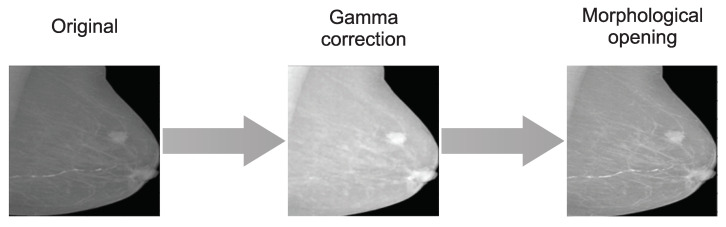
Changes in contrast of original dataset with data augmentation.

Also, a morphological opening with a circular structuring element of radius five was applied in a second augmentation. This operation aimed to increase the local values within the region of the structuring element, allowing an increase in luminance but preserving the morphology of the regions and avoiding outlier values. Figure 2 depicts the resulting contrast augmentation images.

### 2.3. Dataset

INbreast is a digital repository of full-field mammograms annotated by experts [23]. It includes diverse lesions with the corresponding binary mask (groundntruth) of reference, density type, and the BI-RADS grade assessed by an expert. There are 107 mammograms containing masses in breast densities from type ACR-A (low density) to ACR-D (high density). Additionally, these samples also are classified with their corresponding BI-RADS grade (BR).

In a mammogram, breast tissue with low density has low grey levels (dark), while dense tissue presents high grey levels (bright); masses could also present high grey levels. Therefore, the contrast between mass and breast density is relevant for its localization. The four ACR types were tested to evaluate the performance of the automatic localization. Data augmentation was performed (Section 2.2) only for the mass location task, obtaining 321 images to consider condition variations in contrast related to different acquisition devices and a balanced dataset. Classes BR-2 to BR-5 were considered for mass classification since BR-1 does not contain masses, and BR-6 has a positive malignancy by biopsy. The distribution of masses per BI-RADS grade in the dataset was 20 for BR-2, 13 for BR-3, 22 for BR-4, and 21 for BR-5.

### 2.4. Mass Location and Segmentation

**Figure 3:**
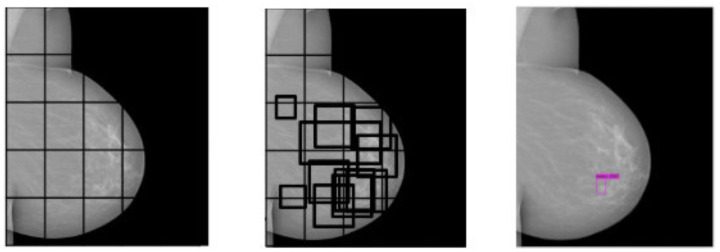
From quadrant analysis (**left**) to similarity estimation among regions (**center**) and the selection of the region with the most significant probability represented in the magenta bounding box (**right**) with YOLOV4.

Since the ROI is focused on the mass, this must be separated from the rest of the breast area. For this task, a pretrained convolutional neural network (CNN) model YoloV4 (You Only Look Once V4) was used for locating the mass area [24], using the configuration parameters of Table 1. In its working process, YoloV4 divides the image into quadrants to identify the ROI, marking regions where a significant similarity is found according to its probability value. Images were resized to 416×416 to improve the online training of the model [25]. As an output, YoloV4 returns the region most likely to contain the object of interest (mass). Figure 3 depicts an example of the general ROI detection process, where the bounding box in magenta indicates the most likely region containing a mass with its respective probability value.

**Figure 4:**
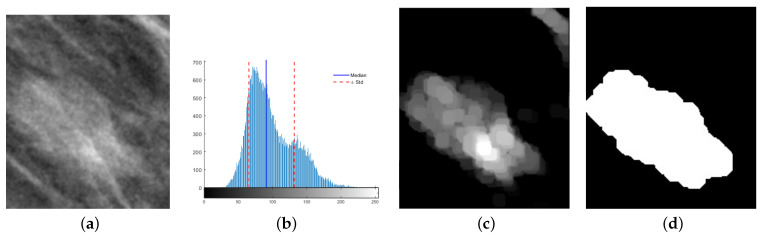
(**a**) The bounding box of a mass detected with YoloV4 and (**b**) the median and standard deviation of its gray-level distribution used as a reference to (**c**) eliminate background and artifacts of the mass; (**d**) an area criterion allows the separation of the most significant region (mass) from the rest to obtain its binary mask.

Although YoloV4 provides the location of the mass indicated within a bounding box, this region also contains part of the breast tissue that is not of interest for the characterization of the mass. A breast mass is high-density tissue associated with high gray levels on mammograms. Therefore, the mass within the bounding box is related to the upper values of the histogram, adopting a slightly skewed Gaussian distribution. The bias depends on the tissue surrounding the mass and usually tends to the left. It was found in the experiments that the central tendency (median) minus one standard deviation allowed the elimination of the pixels mainly associated with the background of the mass. When followed by a morphological opening, small artifacts were also eliminated, as shown in Figure 4c. The area of the remaining regions in the image was estimated, and since the bounding box centered in the mass, only the larger object was preserved. After a simple threshold, the binary mask of the mass was obtained (Figure 4d), helping to analyze its characteristics further.

### 2.5. Mass Characterization

Digital descriptors were computed to find the quantification that expresses a relationship with the qualitative description of the BI-RADS characteristics in mass characterization.

#### 2.5.1. Shape Descriptor

The shape descriptor seeks to relate and quantify the characteristics associated with a probability of malignancy. For instance, a round mass is less likely to be malignant than an irregularly shaped mass. The shape was described based on the mass skeleton *S*, calculated through thinning, identifying its endpoints and bifurcations. First, distances from the endpoints to the center of the skeleton were obtained as the set $p=\left\{d_{1}\left(c,p_{1}\right),d_{2}\left(c,p_{2}\right),\cdots ,d_{k}\left(c,p_{n}\right)\right\}$. Afterward, the asymmetry, average, kurtosis, and variance measures were calculated for *p*. In addition, the endpoints were related to the number of mass lobes associated with the irregularity of the shape (IS) mass with Equation (2), where $N_{p}$ and $N_{b}$ are the numbers of endpoints and the number of bifurcations, respectively.

(2) $$IS=N_{p}+N_{b}$$

**Figure 5:**
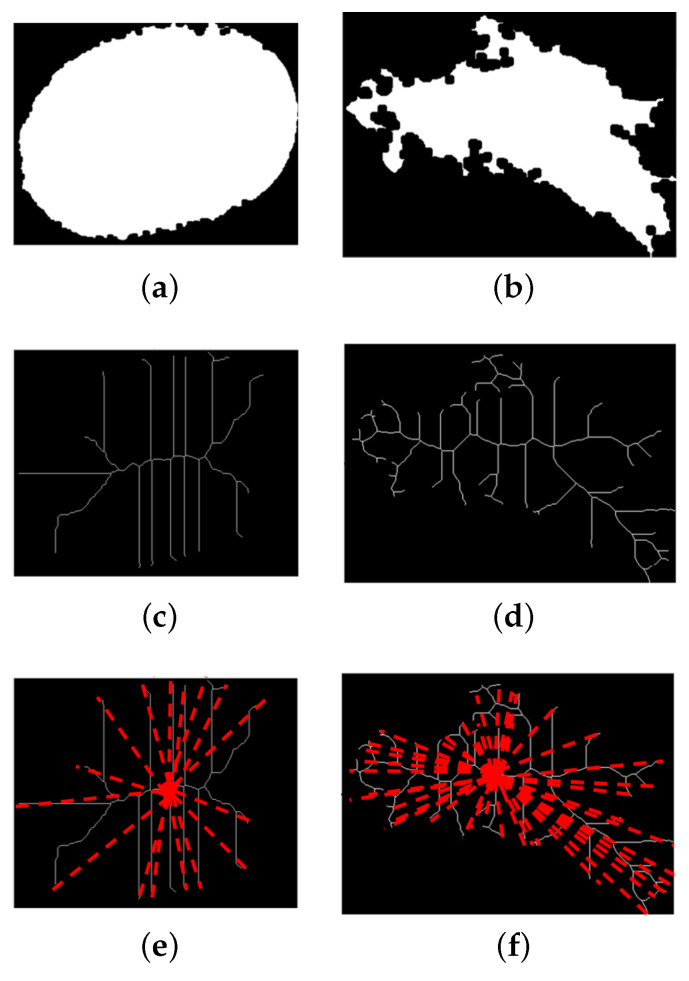
(**Top**) Binary masks of (**a**) benign and (**b**) malignant masses, (**c**,**d**) the simplification of their shape by a skeleton, and (**e**–**f**) their corresponding estimated distances to each endpoint considered for the IS descriptor.

An example of how $N_{p}$ and $N_{b}$ are relevant in the shape description can be seen in Figure 5a, which corresponds to the binary mask of a BR-3, suggestive of benignity, and has an oval shape. The skeleton of the mass (Figure 5c) can be observed with equidistant symmetric branches with endpoints and bifurcations. It can be observed that the number of endpoints and bifurcations is reduced in a regular shape (Figure 5e). On the other hand, Figure 5b shows the binary mask of an irregularly shaped mass of a BI-RADS 6 type with a confirmation of malignancy. The skeleton in Figure 5d shows a higher number of branches, increasing the number of endpoints and bifurcations (Figure 5f). Also, the distances from the center to the endpoints show more significant variation. Therefore, IS and *p* allow the degree of malignancy to be evaluated by associating the number of endpoints and the dispersion of their distances with the regularity of the mass shape.

#### 2.5.2. Margin Descriptor

The margin descriptor provides information about how clearly defined the contour of a mass is. For example, contrary to a speculated one, a continued contour with soft curvature relates to a lower probability of malignancy. A roundness measure could be linked to the margin description of the mass. Three metrics were tested since some could be more sensitive to changes than others. The mean roundness (MR) considers the radius from the center of the border to each pixel on it, as shown in Equation (3), where *n* is the number of pixels in the border, $r_{j}$ is the radius to the *j*-th pixel, and $\bar{r_{b}}$ is the average radius. In contrast, the radius ratio (RR) (Equation (4)) considers the longest ($r_{bmin}$) and shortest radii ($r_{bmax}$). MR and RR showed more sensitivity to changes in the margin to determine how round the shape was. Therefore, they could be adequate for detecting differences among round, lobulated, and speculated masses [26].

(3) $$MR=\frac{1}{n}\sum_{j=1}^{n}\frac{\bar{r_{b}}}{|r_{j}-\bar{r_{b}}|+\bar{r_{b}}}$$

(4) $$RR=\frac{r_{bmin}}{r_{bmax}}$$

The MOR measure [27], based on the radii of the circle in which an object is circumscribed, assesses the degree of regularity of a shape. This descriptor has helped identify objects with slight shape differences [28,29]. It is computed, based on the probability distribution of the radius f(r) from the center of the object to each point of its contour, as the ratio between the area centered in the highest probability f(r) with the deviation of its local minima $k_{1}$ and $k_{2}$ and the total area (Equation (5)).

(5) $$MOR=\frac{\int_{k_{1}}^{k_{2}}f\left(r\right)dr}{\int_{-\infty }^{+\infty }f\left(r\right)dr}$$

#### 2.5.3. Density Descriptor

A mass has a shiny appearance on mammography and must be analyzed with the surrounding breast tissue since their relationship is relevant in malignancy assessment [5]. Although there is no specification of the extent of the surrounding area to be analyzed, breast tissue varies in distribution from dense to fatty. This area must represent the local condition in which the mass develops. Therefore, 20% of the bounding box dimensions were taken as a reference to delimit the area surrounding the mass.

A density map [30] is a representation that allows distinguishing the density differences in tissue independent of the gray-level range of the image and is obtained with Equation (6), where *I* is the original image; *H* is the homogeneity value computed for the subregion centered in I(x,y), comprising the pixels within a defined distance (usually 1), from which the co-occurrence matrix and its probabilities p(i,j) are computed for *N* gray levels [31]. Homogeneity identifies whether a region has high or low variation in its gray levels.

(6) $$DM\left(x,y\right)=I\left(x,y\right)H\left(I\left(x,y\right)\right)=I\left(x,y\right)*\sum_{i=1}^{N}\sum_{j=1}^{N}\frac{p(i,j)}{1+{\left(i-j\right)}^{2}}$$

The density map helps differentiate tissue, highlighting the variation between the region of interest (mass) and the surrounding tissue. Low values are associated with fatty tissue, increasing as the tissue became more dense. Therefore, the scale of DM was divided into four classes using C-means clustering with an associated value *c*: fatty tissue (c=1), low (c=2), medium (c=3), and high density (c=4). Also, C-means was applied in the gray scale of the original image, aiming to differentiate the tissue directly; similarly, a vector with four classes was obtained. Based on the density classes, both representations were considered for describing mass density (Figure 1).

With the help of the binary mask (Figure 4d), a vector with pixels occurring in these classes of DM is obtained for the mass and its surrounding area, helping to describe the BI-RADS density characteristic. The representative value associated with each region depends on the class *c* with the maximum occurrence. Then, the class of mass $c_{m}$ and the class of its surroundings $c_{s}$ are compared, considering the class with a higher value to describe the relationship between the density of the mass and its surroundings ($dms=\max\{c_{m},c_{s}\}$). Low density indicates that the mass mainly comprises pixels of a class lower than the class of its surrounding ($c_{m}<c_{s}$), i.e., a higher occurrence of dense classes outside the mass, and the sample is labeled as dms=1. On the contrary, a high density implies that the class concentrating most of the pixels within the mass is higher than the class outside the mass ($c_{m}>c_{s}$); therefore, dms=3. An equal density (dms=2) suggests a similar predominant distribution inside and outside the mass ($c_{m}≃c_{s}$) considering a difference of ≤10%. Fat-containing density was not considered because there were no samples of this type.

**Figure 6:**
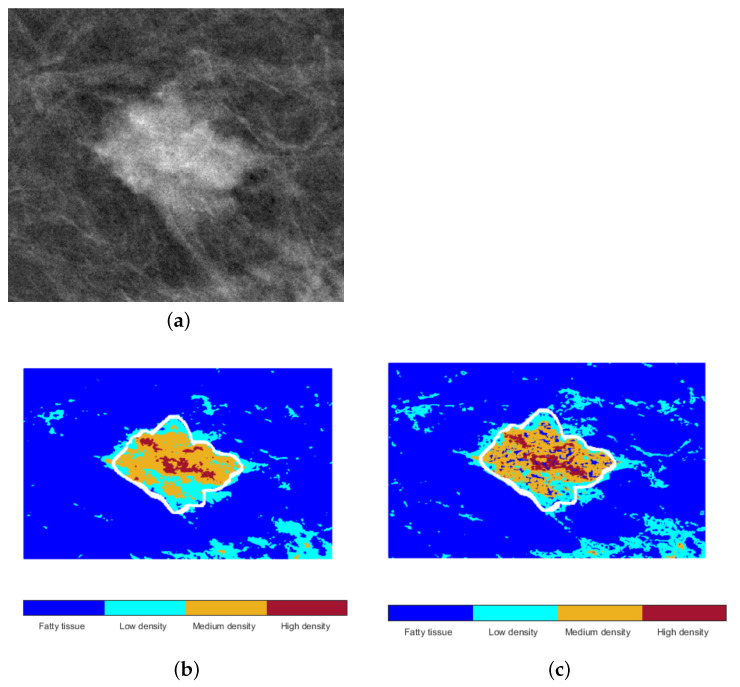
(**a**) Example of an ACR-C (50–75% dense) mass and the separation of fat and dense tissues by C-means over its (**b**) gray-level and (**c**) density map representations. The white line corresponds to the mass marker.

Consider, for instance, the mass in Figure 6a. Clustering performed over gray levels was shown to distinguish the different dense tissues from the fatty tissue (Figure 6b). However, this representation tended to normalize close regions with small changes under the same class. The density map was more sensitive since it considered the variations among near pixels, noticed in the inner of the mass, showing that it was not entirely dense (Figure 6c). This difference could be relevant when fatty and dense tissues are widely mixed in a region. In this case, both descriptors matched when describing a highly dense mass with respect to its surroundings. 

### 2.6. Mass Classification

After locating the mass, the next step is to classify it into one of the BI-RADS categories. A multilayer perceptron (MLP) is a fully connected feed-forward artificial neural network. MLP has at least three layers of nodes: the input, hidden, and output layers. MLPs are trained using the backpropagation technique [32]. The connections within the MLP are randomly initialized and then progressively adjusted based on the available data. These data are input as a vector into the input layer and distributed to the hidden layers to help the network learn to recognize patterns in the training dataset. MLPs are versatile and effective for classification tasks, often achieving impressive performance [33]. They are relatively straightforward to implement, and their tuning parameters have been extensively studied. Table 2 presents the configuration parameters used for the MLP.

### 2.7. Feature Selection

**Figure 7:**
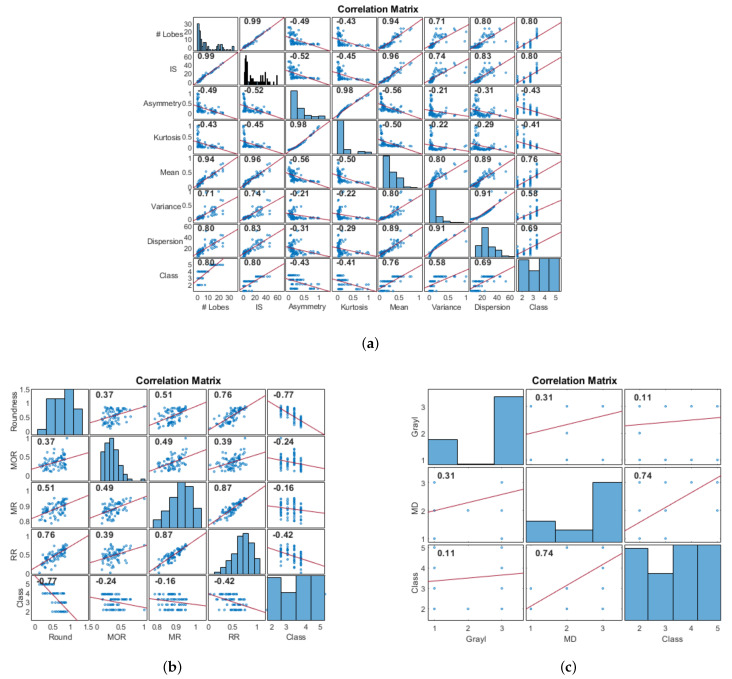
Correlation values between the proposed (**a**) shape, (**b**) margin, and (**c**) density characteristics and the BI-RADS classes. The dots indicate the sample values for each characteristic, and the line indicates the dispersion of the samples around a positive/negative correlation.

After obtaining the region containing the mass and its segmentation, the following process is proposed to obtain a description based on the BI-RADS. The mass characteristics described in Section 2.5 were extracted and analyzed to obtain the relevant ones. First, the BI-RADS shape description was quantified through the number of lobes, irregularity (IS), and distances from the endpoints of the lobes to the center (*p*); from the *p* set, we also computed the measures of kurtosis, average, variance, and standard deviation. Correlation analysis determined the relevance of these characteristics with the BI-RADS class of each mass, as shown in Figure 7a. This analysis aimed to find those characteristics better related to the changes in shape that a mass presents depending on how likely it is to be malignant (BI-RADS class). According to the analysis, the number of lobes, IS, average, and standard deviation of the endpoints were the most correlated to the BI-RADS class.

The margin evaluation is associated with the malignancy of the masses through the continuity or variations in the mass contour. It could be detected by measures related to circularity with different sensitivities to variations in the margin of the shape (Section 2.5). The correlation matrix in Figure 7b shows that the highest correlation with the BI-RADS class occurs with the roundness metric. The negative correlation indicates that a higher roundness value is associated with low-malignancy classes in the standard BI-RADS, coinciding with what the standard describes (Figure 1).

Regarding density, the samples were labeled with their more likely BI-RADS class according to the information extracted from the representations based on DM and gray levels, as described in Section 2.5. It was assumed that the variations in values in both representations could provide relevant information about the environment where the mass had developed. The correlation analysis of the variables showed that DM is a more suitable description for the density relationship inside and outside the mass (Figure 7c).

## 3. Results

The distributions of the dataset described in Section 2.3 for mass location and classification were used to carry out the experiments. A 10-fold cross-validation was applied for mass detection, considering a 90/10 dataset division for training and testing, respectively, and using YoloV4 (Section 2.4). The general performance in mass detection reached a 90±0.08 precision and 91±0.07 of recall. Table 3 shows the percentages of correctly detected masses per density class. Also, the overlap between a predicted bounding box and the bounding box of the ground truth reached 0.90±0.05 according to the IoU value. Once the bounding box containing the mass was obtained, the segmentation of the mass (Section 2.4) was applied, obtaining a Dice score index of 0.83 when compared with the mark of the expert. Therefore, the performed mass location method was suitable for continuing the mass description and classification processes. Based on the proposed characterization (Section 2.7) and MLP with 10-fold cross-validation, the classification per BI-RADS class was performed. The metrics commonly used in machine learning for medical applications, including accuracy, recall, and F1 score, were computed to assess the results [34].

**Figure 8:**
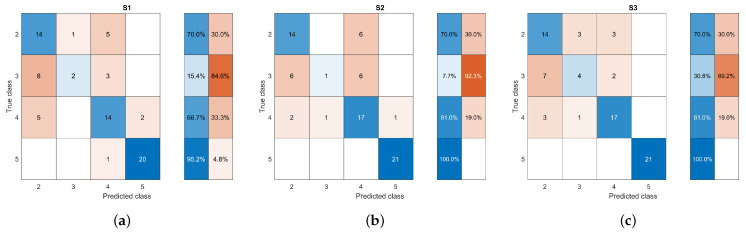
Confusion matrices for classification experiments based on shape descriptors (**a**) S1, (**b**) S2, and (**c**) S3. Blue and orange indicate the correct and incorrect classified sample percentages according to their intensity.

Some shape tests were performed to measure how suitable these characteristics could be for mass classification (Table 4). The first test (S1) used all the initial features, achieving an accuracy of about 0.83 and F1 score of about 0.79, and it was taken as the starting point for comparing whether feature selection helped classification. The second test (S2) considered the four features with the highest correlation, achieving an accuracy of 0.85 and F1 score of 0.83. When masses of different shapes were analyzed, it was found that a circular-shaped mass could be differentiated from the other shapes by a low variance of its distances. However, an oval, lobular, and irregular mass could have similar variances and dispersions of distances, although distributed differently. Then, the last test (S3) omitted the average, variance, and standard deviation. The result showed an increased accuracy (0.87) and F1 score (0.84), achieving the best general performance. Therefore, the number of lobes and IS were used for the BI-RADS shape description. In addition, the confusion matrices were also analyzed to understand how these descriptors were related to the individual identification of classes. Figure 8 shows that the shape descriptors consistently differentiated extreme classes BR-2 and BR-5 in the three experiments. Also, reducing the descriptors in S2 helped to increase the identification of BR-4 samples. However, the shape description by itself could not easily identify BR-3.

**Figure 9:**
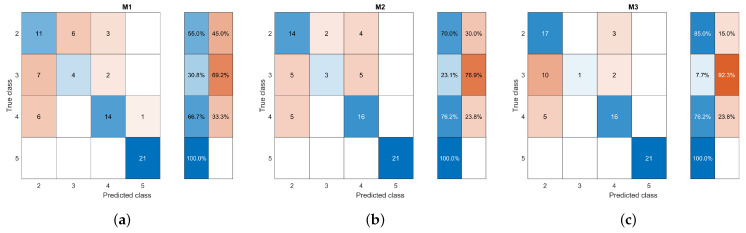
Confusion matrices for classification experiments based on margin descriptors (**a**) M1, (**b**) M2, and (**c**) M3. Blue and orange indicate the correct and incorrect classified sample percentages according to their intensity.

Table 5 shows the performance obtained based on the four circularity measures. It was found that using the four circularity measures (M1) resulted in poor classification performance with an accuracy and an F1 score of about 0.83 and 0.74, respectively. A significant improvement was reached when only the roundness feature was used, reaching an accuracy of 0.85 and an F1 score of 0.81. It was found that margin irregularity, also associated with spicules, is one of the characteristics related to the malignancy of masses. The spicules are particularly difficult to segment as they comprise narrow regions that can stray significantly from the main area of the mass (Figure 5). This behavior could be why the roundness characteristic was not enough by itself to describe the margin of the mass. Therefore, it was complemented by combining it with the number of lobes of the mass, which had already been identified as relevant, and the roundness measure (M3). The outlined combination achieved an improved classification performance of 0.86, providing evidence of the assumption made. The confusion matrices in Figure 9 show the tracking of how the classification improved when using the descriptors in each of the tests.

**Figure 10:**
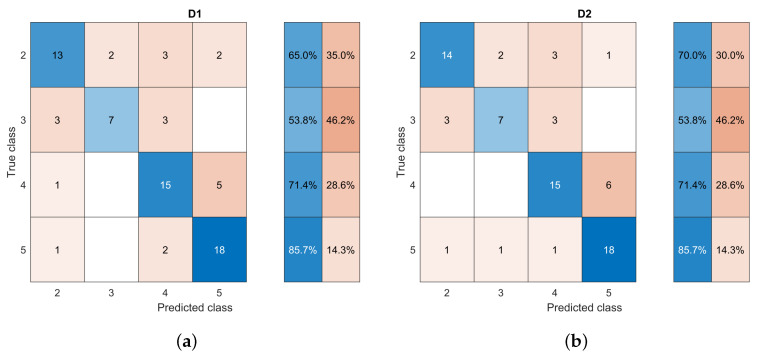
Confusion matrices for classification experiments based on density descriptors (**a**) D1 and (**b**) D2. Blue and orange indicate the correct and incorrect classified sample percentages according to their intensity.

Table 6 presents the evaluation of the results using gray levels and DM for density description (D1) and only DM (D2). Although both descriptions reached similar performance in terms of metrics and confusion matrices (Figure 10), DM was shown to be better correlated with the description.

In previous tests, classes BR-2 and BR-5 were observed to have higher classification rates since extreme classes usually show significant differences; therefore, the classifier could separate them effectively. The characteristics of the masses in classes BR-3 and BR-4 are more similar, which is reflected in a lower classification rate with respect to extreme classes. Moreover, BR-3 is the class with a higher unbalance, which caused poor performance in its classification.

**Figure 11:**
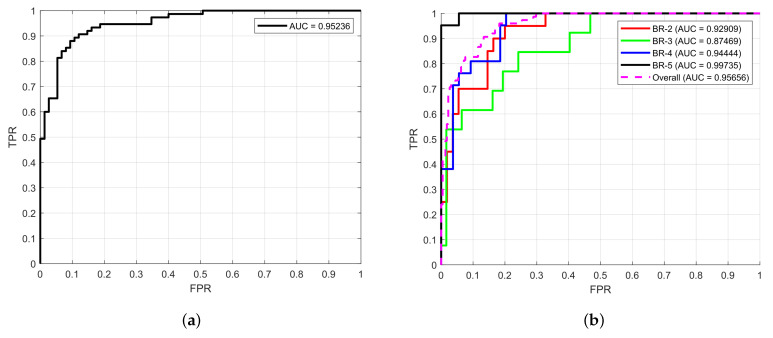
ROC curve for (**a**) binary and (**b**) BI-RADS grade classification.

Now, the aim was to evaluate the complete description for classifying masses according to the BI-RADS using the specific descriptors previously assessed and selected in the experiments for shape (S3), margin (M3), and density (D2). The experimental hypothesis was that using this set of features as a descriptor for mammography mass classification could lead to competitive results compared to the current state of the art. Since the most common breast mass classification is binary (benign/malignant), the description was also tested under this approach. Table 7 shows that an accuracy of 0.90 and F1 score of 0.88 were achieved in the binary classification. For a classification per the BI-RADS grade, those values correspond to 0.91 and 0.85, allowing a more specific and direct identification. This provides an advantage since a binary classification could be vague, e.g., indicating malignancy grades BR-4 to BR-6 under the same label when differences among them could be broad and significant. The area under the ROC curve (AUC) was also computed, obtaining 0.94 and 0.95 for binary and grade classification, respectively (Figure 11).

The classification metrics in Table 4, Table 5 and Table 6 show that using individual features led to high variability, limiting performance regarding the BI-RADS grade. However, combining features enhanced the metrics significantly and reduced the dispersion for both the BI-RADS classification and the binary case. Notably, the recall results for the BI-RADS classification exhibited high dispersion due to the imbalance in the BR-3 class, where the model needed more examples to learn the patterns, leading to higher error rates. To complement the information on the general performance of the classification, balanced accuracy ($a_{B}$) was calculated. $a_{B}$ is a metric defined as the mean of the sum of sensitivity and specificity, specifically focused on imbalanced sets [35]. In this case, class BR-3 showed an imbalance with respect to the other classes. A value of 1 indicates perfect classification, and 0.5 indicates random guessing. The obtained values of $a_{B}$ were 0.87 and 0.85 for the binary and BI-RADS grade classifications, respectively.

**Figure 12:**
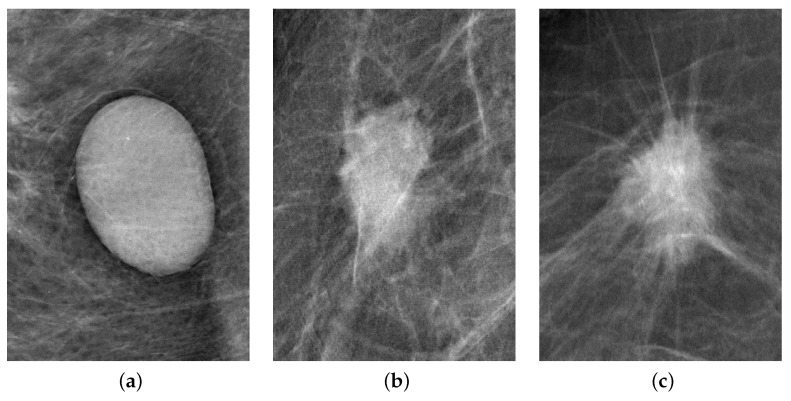
Examples of masses graded as (**a**) BR-2, (**b**) BR-4, and (**c**) BR-5.

Three distinct cases illustrate how selected characteristics effectively classify breast masses based on BI-RADS descriptors, offering a quantitative assessment that enhances clinical diagnosis. Figure 12a depicts a BR-2 case with a notable high roundness, reflecting a regular shape and margin, and low density, characteristics associated with a more likely benign mass. On the other hand, for cases more likely malignant BR-4 and BR-5 (Figure 12b,c), the roundness decreases, suggesting a more irregular shape, the number of lobes increases and the density of the masses increase. The relationship of these three cases with the values of the previously identified characteristics are outlined in Table 8, highlighting the nuances in their variation across different BI-RADS categories.

## 4. Discussion

Diverse approaches have been used to classify breast masses on mammogram, ranging from specific classification methodologies to complete processing systems (Table 9). Dhungel et al. [36] proposed a methodology for location to classification based on deep learning models, proposing a CAD system that works with minimum user interaction and reporting an accuracy of 0.91 for binary classification on the INbreast dataset. However, the F1 score and AUC values were significantly reduced to 0.76. Zhang et al. [37] used the local-invariant characteristics from an MR8 bank filter of the first and second derivatives and a convolutional neural network (CNN) to perform mass classification despite the differences in image conditions. The method was tested on CBIS-DDSM, and the classification results were obtained with a vector of 1024 features, achieving an accuracy of 0.94 and an AUC of 0.97. Lbachir et al. [16] developed a CAT system spanning from location to classification of breast masses. The best classification accuracy was obtained with SVMs (support vector machines) and the MIAS (Mammographic Image Analysis Society) database, reaching a value of 0.94 with a binary approach.

Sparse approximations have also been used for breast mass classification using trained dictionaries (basis signals) [18]. Spatially localized ensemble sparse analysis (SLESA) takes individual blocks of interest (mass) and determines the class through a similarity criterion. An accuracy of 0.90 was obtained with the MIAS database performing 30-cross-fold validation, reaching an F1 score of 0.93. In a later advance [38], deep learning was incorporated for the generation of dictionaries in deep feature SLESA (DF-SLESA), which, combined with SVM, reached 0.72 and 0.77 in accuracy and AUC, respectively. Recently, Singh et al. [17] performed an exhaustive feature computation, aiming to find the most relevant features for breast benign/malignant mass description on the INbreast dataset. Several selection algorithms considered and evaluated textural and shape features until the best performance was reached with a description based on nine features and the k-NN classifier with an accuracy of 0.90 and a sensitivity of 0.92.

The proposed method was competitive with other methods under a similar binary classification approach. However, two relevant aspects must be considered. First, the binary classification could be helpful in a general evaluation, but the classification of interest in clinical practice is related to the BI-RADS grading. The binary classification groups two or more BI-RADS grades into general benign and malignant classes. Nevertheless, some classes present significant differences, mainly in the higher grades, such as BR-4 and BR-6, which often are put together. The second matter relates to the characteristics taken into account for binary mass classification. The compared methods perform automatic feature computation and selection without considering if such features are related to the observations of the expert. Some studies have addressed the complexity of this issue, finding that some selected characteristics of the image may be intrinsic to the DL models but not recognized or explainable by the human eye [39]. Another study conducted specifically for mass classification in mammography [40] reported an analysis of several DL models and how these can have a significant generalization error by overfitting the classification model. The study also mentioned that extracting a large amount of features could generate a feature leakage problem, generating highly optimistic results.

Hence, it is important to base the characterization of masses on the BI-RADS reference standard, which is used by most radiologists to interpret mammograms. The provided description outlines the characteristics of breast masses, focusing on their shape, margin, and density. It demonstrates the ability to differentiate between specific cases and aligns with standard clinical parameters. As a result, it offers a more detailed and objective assessment of breast mass characteristics across various risk levels, which can aid in clinical decision making. This is the first description that employs a BI-RADS grading approach for classifying breast masses, allowing for a direct correlation between the description and expert analysis.

The analysis provides an initial basis for exploring new descriptions that align with the medical community’s reference standard for evaluating breast masses. It was also shown that digital image descriptors can be related to the characteristics of the masses, allowing their quantitative evaluation. Although the results are encouraging, this study was limited to a the INbreast dataset. Therefore, it is imperative to continue testing and refining this approach to evaluate its generalization on more extensive datasets that better represent the wide variability in cases occurring in the real world.

## 5. Conclusions

Automatic breast mass analysis could speed up the mass identification process and provide complementary information to the expert related to breast density. However, it requires the description obtained by automatic methods to be closely related to medical standards. The proposed description is based on the characteristics established by the BI-RADS standard for identifying masses per grade. The results showed that this description allows for identifying the BI-RADS grade of breast masses with a performance close to current methods and that is directly related to the characteristics analyzed by experts. Thus, competitive results were obtained in binary and per-grade classification, with a short description of well-identified features and classic machine learning, making the data explainable.

While this report provides a foundational understanding of the performance of a simple description, it also highlights the potential impact of further research. Testing on a dataset with broader variability, closer to what is observed in clinical practice, could significantly improve the current limitation of training, enhancing the classification and reducing dispersion in the performance of the model. Moreover, exploring a more extensive set of features could lead to a breakthrough in search performance while maintaining interpretability. In future work, we intend to test explainable deep neural networks, extending the identification of other characteristics related to each BI-RADS category.

## Figures and Tables

**Table 1:** Configuration parameters used for YoloV4.

Parameter	Values
ANN size	416 × 416
Batch	64
Max_batches	4000
Steps	5400
Filters	18
Subdivisions	16
Image size	416 × 416
Classes	1

**Table 2:** Configuration parameters used for MLP.

Parameter	Value
Number of layers	2
Neurons per layer	10
Activation function	ReLU
Learning rate	0.001

**Table 3:** Samples per density class after data augmentation of the INbreast database [23].

Breast Density	Total Samples	Mass Location (%)
A	126	80.0 ± 4.8
B	114	94.4 ± 3.2
C	63	90.4 ± 14.2
D	21	85.7 ± 7.7

**Table 4:** Performance of relevant characteristics in shape description for mass classification.

Test	Accuracy	Recall	F1 Score
S1	0.83 ± 0.09	0.62 ± 0.31	0.79 ± 0.20
S2	0.85 ± 0.09	0.65 ± 0.38	0.83 ± 0.17
S3	0.87 ± 0.08	0.71 ± 0.27	0.84 ± 0.11

**Table 5:** Performance of relevant characteristics in margin description for mass classification.

Test	Accuracy	Recall	F1 Score
M1	0.83 ± 0.11	0.64 ± 0.27	0.74 ± 0.18
M2	0.85 ± 0.09	0.67 ± 0.31	0.81 ± 0.15
M3	0.86 ± 0.09	0.67 ± 0.39	0.86 ± 0.16

**Table 6:** Performance of relevant characteristics in density description for mass classification.

Test	Accuracy	Recall	F1-Score
D1	0.85 ± 0.03	0.70 ± 0.10	0.77 ± 0.04
D2	0.85 ± 0.02	0.70 ± 0.12	0.78 ± 0.07

**Table 7:** Classification performance based on the proposed mass description per class and in general.

Classification	Accuracy	Recall	F1 Score
BI-RADS grade	0.91 ± 0.05	0.80 ± 0.19	0.85 ± 0.10
Binary	0.90 ± 0.10	0.89 ± 0.05	0.88 ± 0.07

**Table 8:** Three clinical cases reported under the BI-RADS standard and represented under the selected set of characteristics.

ID	# of Lobes	IS	MR	DM	Bi-RADS
22678787	0	2	0.90	Low	2
22670324	3	6	0.55	Medium	4
20587612	14	26	0.34	High	5

**Table 9:** Comparison of results of breast mass classification.

Report	Classification	Dataset	Method	Accuracy	Recall	F1 Score	AUC
[36] (2017)	Binary	INbreast	CNN	0.91 ± 0.02	0.98	0.76	0.76 ± 0.23
[37] (2020)	Binary	CBIS-DDSM	CNN	0.94	0.89	–	0.97
[16] (2020)	Binary	MIAS	SVM	0.94	–	–	0.95
[18] (2021)	Binary	MIAS	SLESA	0.90	–	–	0.93
[17] (2022)	Binary	INbreast	k-NN	0.90	0.92	–	–
[38] (2023)	Binary	MIAS, CBIS-DDSM	InceptionV3-SLESA	0.72	–	–	0.77
Proposed	Binary	INbreast	MLP	0.90 ± 0.10	0.89 ± 0.05	0.88 ± 0.07	0.95
Proposed	BI-RADS	INbreast	MLP	0.91 ± 0.05	0.80 ± 0.19	0.85 ± 0.10	0.95

–: Not reported.

## Data Availability

The original contributions presented in this study are included in the article. Further inquiries can be directed to the corresponding author.
